# Bartsocas-Papas Syndrome: Unusual Findings in the First Reported Egyptian Family

**DOI:** 10.1155/2011/428714

**Published:** 2011-11-02

**Authors:** E. M. Abdalla, H. Morsy

**Affiliations:** Department of Human Genetics, Medical Research Institute, Alexandria University, 165 El-Horreya Avenue, El-Hadra, Alexandria 21561, Egypt

## Abstract

Bartsocas-Papas syndrome (BPS) is an autosomal recessive syndrome with severe craniofacial, limb, and genital abnormalities. As of 2011, 24 published cases and families were registered in the Orphanet Report Series. Compared to other disorders characterized by pterygia, the condition is usually more severe and often lethal: most affected patients die in utero or shortly after birth. We report the first Egyptian family with Bartsocas-Papas syndrome comprising three cases; our proband who was a female infant with severe craniofacial and limb anomalies typical of Bartsocas-Papas syndrome, a similarly affected female fetus which died in utero at the 7th gestational month, and a 16-year-old mentally retarded uncle who presented with some of the typical features of Bartsocas-Papas syndrome, including syndactyly, thumb hypoplasia, and microphthalmia. This male patient actually did not present with pterygia, however, we find his clinical description noteworthy.

## 1. Introduction

Bartsocas-Papas syndrome (BPS, OMIM 263650) is a rare autosomal recessive syndrome characterized by severe popliteal webbing, ankyloblepharon, syndactyly, orofacial clefts, filiform bands, hypoplastic nose, and ectodermal anomalies [1]. Most affected cases die perinatally or few months after birth [2], although occasional survival has been reported [3, 4]. Since its first description in 1972 [1], BPS has been diagnosed repeatedly in literature with 24 cases reported up till now, mostly of Mediterranean ancestry [5, 6]. In 1998, Massoud et al. [2] reported the first Arab family with BPS. Here, we report the first family from Egypt with two typical cases and some unusual interesting presentation in the uncle case.

## 2. Case Presentation

### 2.1. Case  1: Affected Living Infant

**Figure 1:**
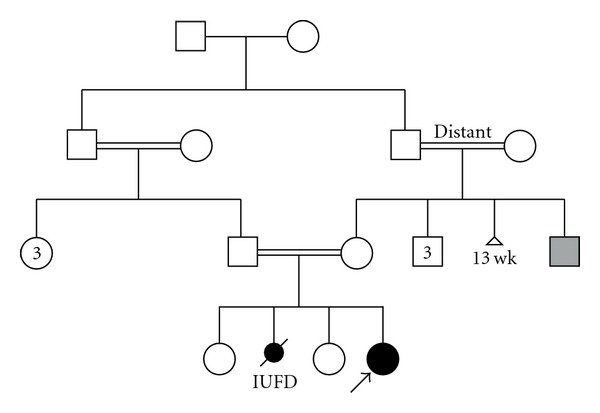
The family pedigree.

A female infant was referred at the age of three months for genetic evaluation of her severe craniofacial and limb malformations. The patient was the third child born to Egyptian healthy consanguineous parents; a 28-year-old mother and a 37-year-old father who had another two healthy daughters (Figure 1). The antenatal history was unremarkable except for weak fetal movement, and there was no exposure to teratogens. The patient was delivered normally, with an average birth weight.

**Figure 2:**
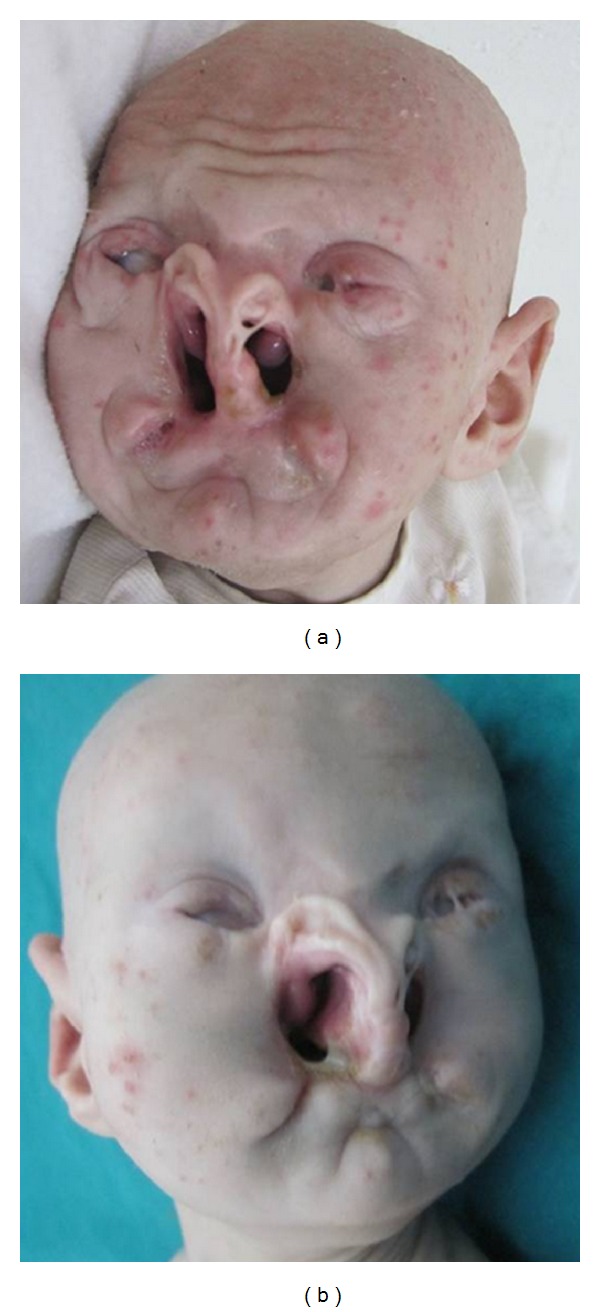
The BPS patient closeup of face showing severe craniofacial anomalies.

On examination, there were striking craniofacial anomalies including severely malformed nose with clefting, major eye anomalies and total absence of eyebrows, eyelashes, and scalp hair. The multiple anomalies of the eyes included microphthalmia, small hazy corneas, left eye ankyloblepharon with filiform bands, multiple right eyelid colobomas, and short palpebral fissure with marked upward slanting. Other abnormalities seen at the orbital region were hypertelorism and peculiar two symmetrical indentations over the nasal root. The nose was severely hypoplastic and distorted with a unilateral synechium extending downwards from the left ala nasi. Although no mouth or lips could be identified, two salivary pits were distinguished at the sites of mouth corners and saliva bubbles were coming out from them. A striking bilateral orofacial cleft extending into the alae nasi and connecting with a bilateral cleft palate was noted. The mandible and maxilla were severely hypoplastic, with a transverse groove over the chin, while the ears were relatively large, cupped, and low set (Figure 2).

**Figure 3:**
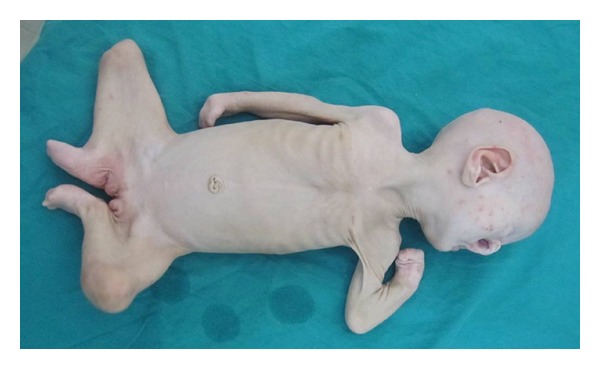
Total view of the proband showing multiple webs at the shoulders, elbows, with huge popliteal webs extending from heel to genitalia, a short sternum and low set umbilicus.

**Figure 4:**
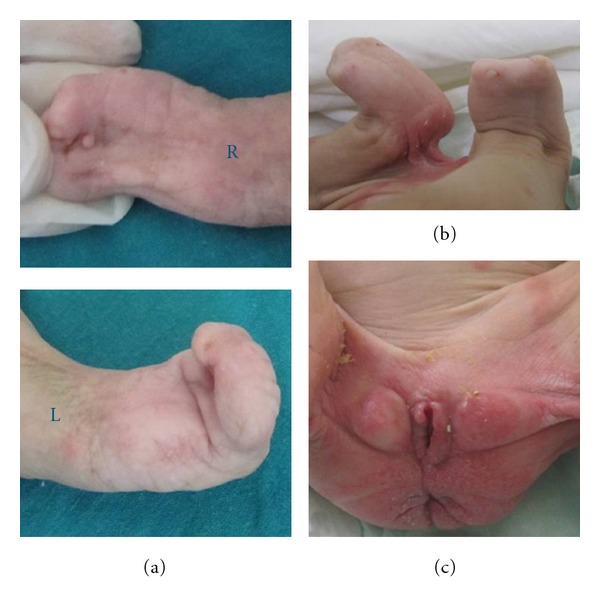
(a) Complete syndactyly (mitten hands), absent nails, left single transverse palmar crease and right tag (b) Adactyly of both feet with a single toenail in right foot (c) Ambiguous external genitalia. Note the stenotic anal orifice.

Major pterygia were found on almost all the joints; shoulders, elbows, wrists, hips, knees, and ankles, whereas the neck was spared. There was a particularly huge thick popliteal webs extending from ischia to heels, severely distorting the lower limbs (Figure 3). Complete syndactyly between all fingers of both hands, flexed fingers, absent thumbs and bilateral single transverse palmar crease were also noted (Figure  4(a)). There was adactyly of both feet with dorsiflexion and pes planus. The nails were absent in all fingers and toes except for one rudimentary nail in each foot. Multiple skin pits were found at dorsal aspect of hands and feet at sites of absent nails, while two pits were found at the palmar surface of hands, with one skin tag at the right palm (Figure 4). 

Other anomalies evident in the patient included a very short sternum, laterally-displaced nipples, low set umbilicus, and prominent veins over chest and abdomen. In addition, five skin tags were observed at the chest (Figure 3). 

The patient had ambiguous external genitalia with absent clitoris. The labia majora formed two empty swellings with overlying corrugated skin, and a skin tag was seen on the labia minora. The anal orifice was stenotic and the iliac and sacral bones were prominent with a sacral dimple (Figure  4(c)). Anthropometric measurements of the patient were all below the third percentile.

Abdominal and pelvic US, echocardiogram, and cranial MRI were requested. Unfortunately, the infant died shortly after examination, however, a venous blood sample was obtained and DNA was kept for future molecular studies. 

### 2.2. Case  2: Affected Fetus Which Died in Utero

The mother experienced a fetal demise 3 years before the birth of the proband. A female fetus with genital ambiguity was delivered after a 26-week gestation complicated by intrauterine growth retardation. The parents described clinical findings similar to the proband; however, there were no medical records or pictures of this affected fetus (Figure 1).

### 2.3. Case  3: Maternal Uncle with Unusual Findings

According to family history, the proband's maternal uncle was mentally retarded and has complete syndactyly between 2 fingers in one hand. He was recalled for genetic evaluation. 

**Figure 5:**
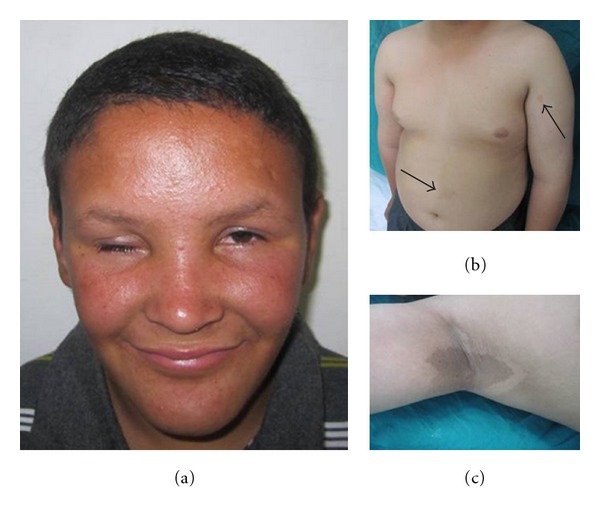
(a) Facial features of the proband's maternal uncle, (b) truncal obesity and multiple café au lait spots. Note large one at the right axilla (c).

Upon attending our clinic, we found the 16-year-old maternal uncle a mentally retarded male with a friendly attitude. On examination, striking ocular, cutaneous and limb anomalies were noted. There was microphthalmia with short and narrow palpebral fissure, sparse lower eyelashes, ptosis of left eyelid and deeply set eyes. He also had an oval face with proganthism, short and smooth philtrum, wide mouth with thin lips, and high arched palate (Figure  5(a)). The patient also had a short neck, widely spaced nipples and multiple café au lait spots with a peculiar large dark one on the right axilla (Figures  5(b) and 5(c)). Truncal obesity and hypoplastic external genitalia were also evident. 

**Figure 6:**
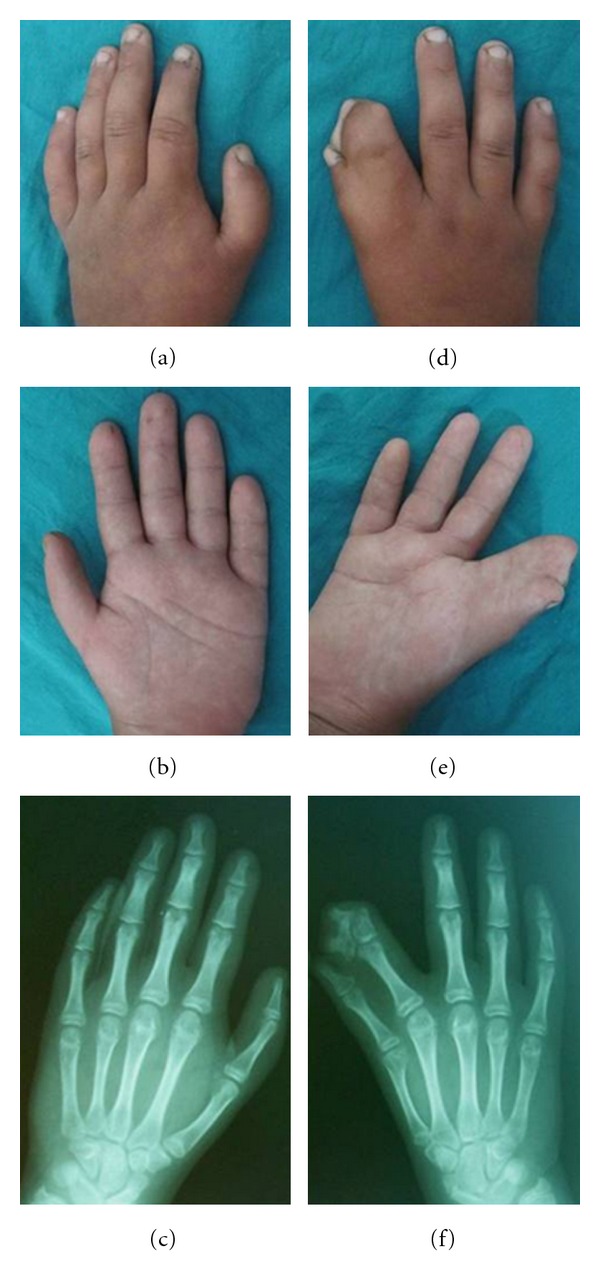
Limb abnormalities in the patient's uncle. Hands photographs and radiographs showing (a)–(c) hypoplastic thumb in the left hand and (d)–(f) syndactyly between the first and second fingers, with an extra nail in the right hand.

Limbs examination revealed a complete thumb-index syndactyly of the right hand with an extra nail in the syndactylous web. The left thumb was short with hypoplasia of the thenar eminence and the right hand creases were distorted (Figure 6). Both feet had short and broad toes, especially affecting the halluces.

The patient's height and head circumference were below the third percentile. Radiographs of hands showed an abnormal hypoplastic trapezoid middle phalanx with duplication of distal phalanx of the right second finger and a hypoplastic first metacarpal in both hands (Figure 6). 

## 3. Discussion

Bartsocas-Papas syndrome (BPS) is a severe autosomal recessive syndrome characterized by neonatal or intrauterine death in most cases, severe popliteal webbing, oligosyndactyly, genital abnormalities, and typical face with craniofacial anomalies [1, 6]. The infant described in this paper had all the clinical features distinctive of BPS [6, 7]. Additional findings previously reported in the literature were also noted in the case such as hyperteloism with upward slanting of palpebral fissure, eyelid colobomas, salivary pits, transverse groove at the chin, widely spaced nipples, short sternum, and stenotic anal orifice [2, 6]. Contrary to the frequent reporting of IUGR [2, 6] and low birth weight in cases of BPS, our case was born with an average birth weight. 

Although the presence of skin tags has been previously described in BPS cases, they were all found at the chest [2, 8]. Here, in addition to five skin tags noted over the chest, other tags were found at the right palm and at the right labia minora; unusual sites that have not been reported in the literature. Other unusual findings include the two indentations noted between eyes. 

The marked popliteal pterygium is the key finding in BPS cases. Besides the huge popliteal webs, our case also showed multiple webs involving the upper limbs; particularly a clinically evident axillary web. Indeed, this finding is not typical of BPS cases as most of the previously reported cases did not have upper extremity webs [9]. Nonetheless, axillary webs were described as an interesting additional finding by Aslan et al. [10] and by Veenstra-Knol et al. [6]. Accordingly, it has been proposed that axillary web may be one of the rare features of this syndrome [9].

The family history in this paper, normal consanguineous parents with two affected babies, is highly suggestive of autosomal recessive inheritance. Thus, we have a case of multiple pterygia with early lethality, that is, most probably, transmitted as an autosomal recessive condition. According to Hall et al. [8], the autosomal recessive limb pterygia syndromes include two lethal forms: the lethal multiple pterygium syndrome (LMPS) and the lethal popliteal pterygium syndrome (LPPS). The terms “lethal popliteal pterygium syndrome” and “lethal pterygium syndrome with facial clefting” have been usually used synonymously to BPS (the same OMIM entry 263650). Another syndrome presented as a different entity in the OMIM catalogue [11]; the multiple pterygium syndrome, Aslan type (OMIM 605203) [10], has a strong resemblance to BPS cases. Interestingly, Teebi et al. [12] and Fryns and Moerman [13] suggested that the disorder reported by Aslan et al. [10] was BPS. We believe that the definite demarcation maybe difficult at this stage and it will not be until the gene for BPS is known that we will be able to split or merge these syndromes appropriately. 

Regarding our patient, even though LMPS is an autosomal recessive condition, the absence of pulmonary hypoplasia, edema and cystic hygroma, or neck webbing, together with the presence of severe craniofacial anomalies excludes its possibility and favors the BPS diagnosis [8, 10, 12]. On the other hand, the localization of the pterygia in our case was more similar to those in popliteal pterygium syndrome (PPS; OMIM 119500) [14]. However, PPS is inherited in an autosomal dominant manner [8, 14]. Furthermore, the lip pits and the pathognomonic nail abnormalities, which are distinctive findings in PPS [14, 15], were not seen in our case. 

The examination of the proband's uncle raised many questions. He had microphthalmia, with short and narrow palpebral fissure, sparse lower eyelashes, hypoplastic thumbs and complete syndactyly, all of which are uncommon dysmorphic features that could be in BPS. However, he did not present with pterygia, the cardinal feature present in the majority of cases of BPS [2, 6]. The case survived to adolescence although it is well known that the BPS is a lethal syndrome with very few case reports of patients who survived beyond infancy [3, 4]. The patient had mental retardation and short stature, two characteristics that were not argued in BPS due to early lethality of cases. In addition, he had multiple café au lait spots, a feature not previously reported in BPS. Despite our exhaustive search in the genetic disorders databases, we could not reach a diagnosis. 

Could it be a mild form of BPS? is It possible that this patient represents the milder end of the spectrum of BPS? However, intrafamilial variation of expression has rarely been reported, and it has never been so extensive [6]. Is it possible that the patient is a heterozygous carrier for the unknown recessive gene of BPS, with unexpected expression of the clinical features? This suggestion has never been raised for BPS and certainly for most of the other autosomal recessive disorders. However, we came across a few literature reports of heterozygote expression in Meckel-Gruber syndrome [16, 17] and multiple pterygium syndrome [18]. Lastly, although we think it is a remote possibility and unusual coincidence, this patient may be affected by a completely different entity. The dilemma of whether this patient represents a mild form of BPS, heterozygote expression, or simply another unknown syndrome, could not be solved based on clinical grounds alone. Hence, the molecular diagnosis following gene identification is highly recommended. 

In conclusion, we report here a family with BPS, the first from Egypt. The intriguing case of the proband's relative who despite lacking the major limb and craniofacial malformations of BPS, shares common features with the syndrome, raised many questions and led to several speculations. This family emphasizes the extreme importance of molecular studies to identify the gene responsible for the BPS. This will answer such unsolved questions, allow better understanding of the pathogenesis of the syndrome and of course will help in genetic counseling and prenatal diagnosis. Finally, we believe that this interesting family will be very useful in the search for the BPS gene and can form a good starting point for tracing such a gene. 

##  Consent

Written informed consents were obtained from the family for publication of this case report and accompanying images. A copy of the written consent is available for review by the journal's Editor-in-Chief.

##  Conflict of Interests

The authors indicate they have no financial relationships relevant to this paper to disclose.

## Abbreviations

BPS: Bartsocas-Papas syndrome
LMPS: Lethal multiple pterygium syndrome
PPS: Popliteal pterygium syndrome
LPPS: Lethal popliteal pterygium syndrome
IUGR: Intrauterine growth retardation.
